# Miscellaneous

**Published:** 1887-02

**Authors:** 


### MISCELLANEOUS.

QUININE.—The action of sulphate of quinine in well-characterized intermittent fever is, when administered in one single dose of fifteen or twenty grains at night, more certainly efficacious than smaller doses at intervals. The single, full dose taken at night is seldom followed by any depressing effects. The usual *tinnitus aurium* (ringing in the ears) observed to follow small doses administered during the day, constitutes one of the great objections upon the part of most persons to take this salt; and the small doses are never so efficacious.—*Progress.*

AN AMPLE LEGACY.—The young men attending the Harvard Medical School, have a predjudice against the female students, one of whom is Miss Annie Copeland, of Bridgewater. They called her to attend a case of fracture of a leg. The patient was a man fifty years old, and when the lady exposed the damaged member she found it to be a broken wooden leg. She sent for hammer and nails, made substantial repairs and charged $25, the collection of which she enforced by the aid of a constable.

TREATMENT OF RINGWORM.—Dr. Searlis recommends oil of turpentine for the cure of ringworm of the scalp (*Medicina Comtemporanea*). The hair should be closely cut over the affected part, and for a short distance around, and then turpentine is to be liberally applied, and rubbed in well with the finger. This is allowed to remain for about five minutes, and is then washed off with carbolic soap, and afterward with hot water, and the patch is then painted with dilute tincture of iodine, or with a two-per-cent solution of iodine in turpentine. The application is to be made once or twice a day, and is not painful, though it causes a slight smarting. The Doctor claims that he has cured in ten days, by this method, cases of ringworm that have resisted all other modes of treatment.

PROF. WILLIAMS relates the following in the *Ind. Electic Journal*: "I once had treated a case of inflamed eyes with finely-powered salt. When the woman asked me what I was using, I said it was chloride of sodium. It worked well for several days. Once, as I was washing it off with water, the patient tasted it. Smacking her lips, she knowingly said: "It tastes like salt." I said: "it is salt." She straightened up, looked me defiantly in the eyes, and said: "What! salting my eyes and charging me two dollars a day for that? I can get them salted cheaper at home." I never saw her again. As long as it was chloride of sodium it worked like a charm. When, suddenly, it became salt, the charm went with my patient.

THE QUALIFICATIONS OF DOCTORS.—People in large cities do not pay sufficient attention to the qualifications of doctors. If a man be sick, the nearest physician is called. That physician, we fear, from personal observation, sometimes kills his patient, because the neighbors are naturally kept from exercising their own common sense in the case. The doctor, having carried his course of treatment to its logical conclusion, pronounces the patient beyond hope, and then only another doctor, generally a good one, is called to attend the man in dying. This latter doctor will say no word concerning the previous treatment.

Now, why? Is the business of doctoring merely an experiment, wherein it is unsafe to say a quack or an empiric has mistreated his man, and unsafe because the learned doctor may do the same thing to-morrow? There is in most folks a desire to put their own family physician on their best friends. Probably, this saves many lives through holding off the beginner in drugs. We would be pleased to see a custom established that would make it possible for a student doctor to accompany a physician of twenty years' experience in his rounds for about two years. An immense amount of money is paid to doctors. When a doctor knows all there is to be known, the money is well spent. When the doctor is compelled to read his books at every case it is apt to be unlucky money. *The Current.*

Looking after the Scalp.—Many think that by cutting the hair short they increase its growth. But this is doubtful. Women rarely become bald; yet they seldom cut their hair as do men. May not their immunity from a shining pate be partly due to the fact that they do not patronize the barber, nor wear tight head-gear? If in early life, our young men would look after their scalps, even while they do not appear to need attention, it might save them the trouble of looking after them in sorrow at a later period, when it will not do much good. If they do not, the time will come when we shall have a race of human beings without hair. —*Boston Budget.*

### PHRENOLOGY.

Phrenology! now look at that!
They've made one's head a riddle,
And say we carry in our hat
A guide to what we should be at,
And where we'd make a fizzle.
Well I should say it's quite enough
To make a fellow sea-sick;
I can't believe the shallow stuff
That shows a bump for taking snuff,
And one for taking physic:
Just see! it tells you how to wed,
And when you should begin it.
It can't be true, whatever's said,
Now, for example, take my my head.
* * * *
Oh, pshaw! there's nothing in it!

